# Applied immuno-epidemiological research: an approach for integrating existing knowledge into the statistical analysis of multiple immune markers

**DOI:** 10.1186/s12865-016-0149-9

**Published:** 2016-05-20

**Authors:** Bernd Genser, Joachim E. Fischer, Camila A. Figueiredo, Neuza Alcântara-Neves, Mauricio L. Barreto, Philip J. Cooper, Leila D. Amorim, Marcus D. Saemann, Thomas Weichhart, Laura C. Rodrigues

**Affiliations:** Instituto de Saúde Coletiva, Federal University of Bahia, Rua Basílio da Gama, s/n - Canela, Salvador, BA 40110-040 Brazil; Mannheim Institute of Public Health, Social and Preventive Medicine, University of Heidelberg, Heidelberg, Germany; Instituto de Ciências da Saúde, Federal University of Bahia, Salvador, Brazil; Centro de Pesquisa Gonçalo Muniz, Fundação Oswaldo Cruz (FIOCRUZ), Salvador, Brazil; Institute of Infection and Immunity, St George’s University of London, London, UK; Centro de Investigación en Enfermedades Infecciosas y Crónicas, Pontificia Universidad Católica del Ecuador, Quito, Ecuador; Instituto de Matemática, Federal University of Bahia, Salvador, Brazil; Clinical Division of Nephrology, Internal Medicine III, Medical University of Vienna, Vienna, Austria; Institute of Medical Genetics, Medical University of Vienna, Vienna, Austria; London School of Hygiene & Tropical Medicine, London, UK

**Keywords:** Immuno-epidemiology, Correlated immune markers, Cytokines, Statistical analysis, Conceptual frameworks

## Abstract

**Background:**

Immunologists often measure several correlated immunological markers, such as concentrations of different cytokines produced by different immune cells and/or measured under different conditions, to draw insights from complex immunological mechanisms. Although there have been recent methodological efforts to improve the statistical analysis of immunological data, a framework is still needed for the simultaneous analysis of multiple, often correlated, immune markers. This framework would allow the immunologists’ hypotheses about the underlying biological mechanisms to be integrated.

**Results:**

We present an analytical approach for statistical analysis of correlated immune markers, such as those commonly collected in modern immuno-epidemiological studies. We demonstrate i) how to deal with interdependencies among multiple measurements of the same immune marker, ii) how to analyse association patterns among different markers, iii) how to aggregate different measures and/or markers to immunological summary scores, iv) how to model the inter-relationships among these scores, and v) how to use these scores in epidemiological association analyses. We illustrate the application of our approach to multiple cytokine measurements from 818 children enrolled in a large immuno-epidemiological study (SCAALA Salvador), which aimed to quantify the major immunological mechanisms underlying atopic diseases or asthma. We demonstrate how to aggregate systematically the information captured in multiple cytokine measurements to immunological summary scores aimed at reflecting the presumed underlying immunological mechanisms (Th1/Th2 balance and immune regulatory network). We show how these aggregated immune scores can be used as predictors in regression models with outcomes of immunological studies (e.g. specific IgE) and compare the results to those obtained by a traditional multivariate regression approach.

**Conclusion:**

The proposed analytical approach may be especially useful to quantify complex immune responses in immuno-epidemiological studies, where investigators examine the relationship among epidemiological patterns, immune response, and disease outcomes.

**Electronic supplementary material:**

The online version of this article (doi:10.1186/s12865-016-0149-9) contains supplementary material, which is available to authorized users.

## Background

**Fig. 1:**
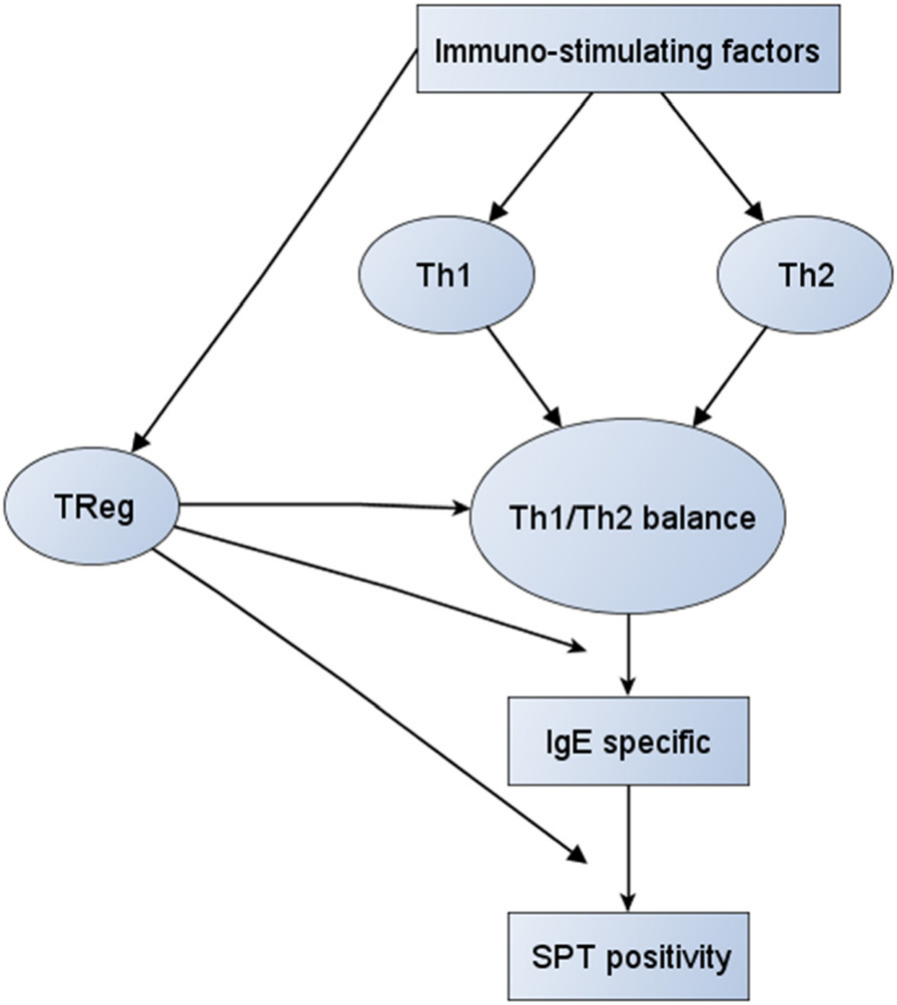
Conceptual model: The figure visualizes current immunological concepts underlying atopic diseases and allergy. First, immuno-stimulating factors, e.g. infections or exposure to dust mite excretion or to helminths exert differential effects on Th1-, Th2 or TReg responses. In concert these mechanisms are presumed to affect the Th1/Th2 balance considered to be a major determinant for regulation of specific IgE that is a predictor for a positive skin pricktest (SPT). Further downstream, the model assumes that T-Reg modifies the effect of Th1/Th2 balance on the regulation of specific IgE antibodies as well as the effect of specific IgE on positivity of skin pricktest (SPT)

Basic and experimental research during recent years has advanced our understanding of immune-regulatory mechanisms in health and disease. An example is the concept of Th1 versus Th2 responses applied in immuno-epidemiological studies [1] aiming to identify causal pathways linking immunological mechanisms to health outcomes such as allergy. To investigate possible pathways in epidemiological studies, researchers regress clinical outcomes or surrogate endpoints to heterogeneous predictor variables collected from large populations. Immuno-epidemiologists typically enumerate immunological mechanisms of interest by collecting various immune biomarkers presumed to result from different immune cells. For example, in allergy research, researchers obtain concentrations of different cytokines presumed to result from Th1, Th2, or T-Reg cells from cell cultures under different immunological conditions. The cytokine measurements are assumed to characterise the major immunological pathways involved in the development of atopic diseases, i.e. the Th1/Th2 balance and immune regulation (Fig. 1) [2–5].

Researchers often have to deal with several peculiarities of such immunological data [6]. These include non-normality of distributions due to substantial skewness or the existence of non-detectable values (“non-detects” are concentrations of a marker below the detection limit of an assay). For each of these problems, statistical approaches have been proposed: robust methods to deal with the non-normality [7], imputation techniques to deal with “non-detects” [8], use of Kaplan-Meier analysis for censored data [9], advanced regression models for non-normal data (Tobit- or Quantile regression) [10, 11], and methods for the analysis of repeated immunological measures [12]. These methodological advances have substantially improved the use of a few immunological parameters, such as outcomes or predictor variables, in classical regression approaches.

However, recent progress in analytical methods has considerably increased the number of immune markers to be collected in immuno-epidemiological studies. Markers are often highly correlated and immunologists have already embraced the appropriate “latent variable” approach by considering a battery of correlated immune markers, like cytokines, as indicators of underlying latent mechanisms (Fig. 1). Previously applied approaches incorporating the idea of latent variables have been predominantly data driven, such as principal component analysis [13, 14] or latent class analysis [15]. Other authors have used simple summary measures of biologically unrelated immune responses, such as ratios of distinct immune responses (e.g. Th1/Th2 ratio) [4]. What is missing is an analytical strategy that additionally incorporates the immunologists’ hypothesised underlying biological mechanisms and uses appropriate and easy-to-follow statistical procedures.

**Fig. 2:**
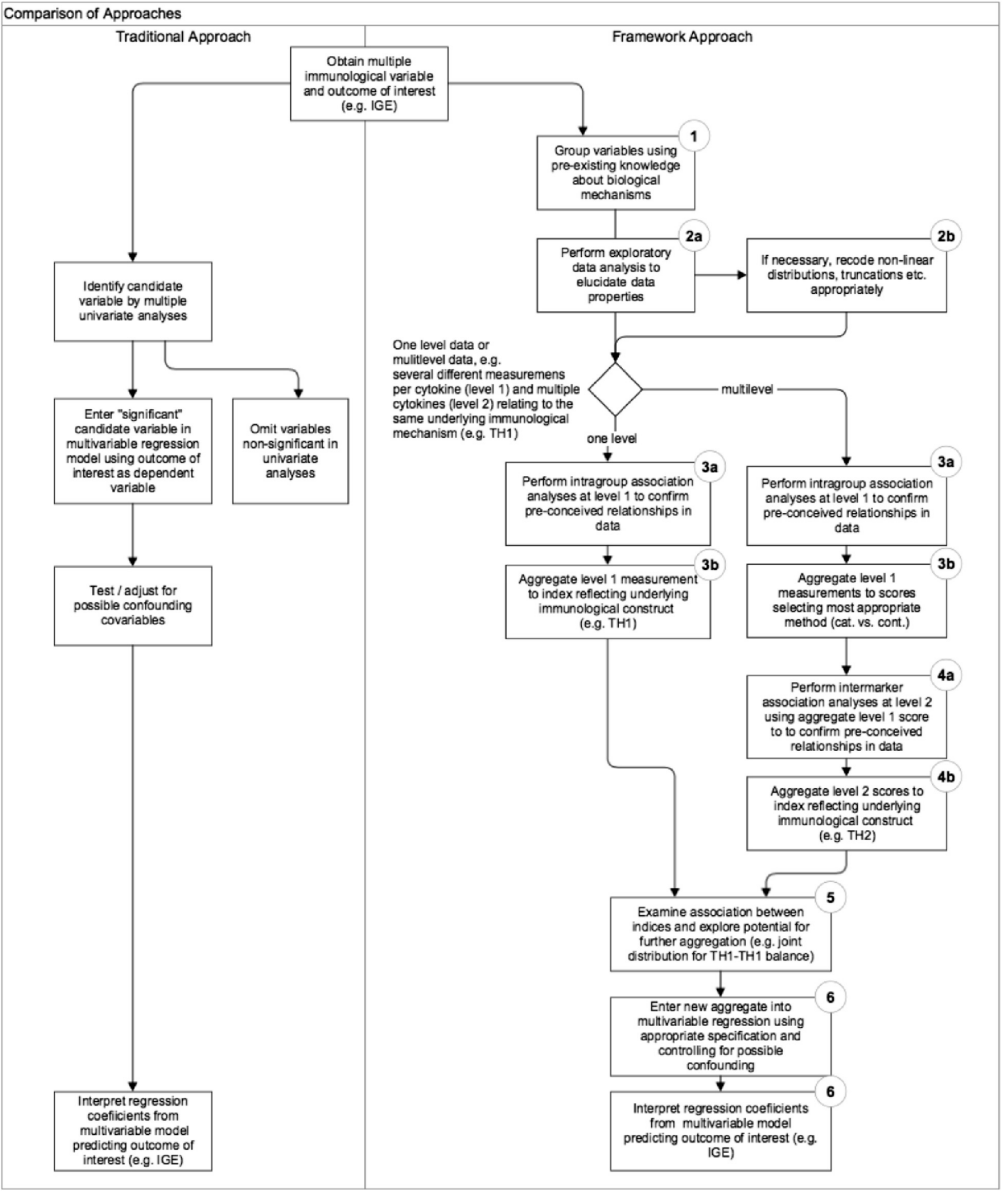
Traditional approach vs. framework approach for statistical analysis of multiple immune markers. The left side illustrates the conventional statistical regression approach selecting potentially relevant markers primarily on the basis of statistical significance in a multivariate regression model. The right side illustrates the proposed approach incorporating a priori existing immunological knowledge (1) in combination with appropriate statistical methods (2-6). The aim is to iteratively aggregate information from single measurements (2) to summary indices reflecting the underlying immunological construct (3-4) either to further aggregate these summary indices to joint distribution constructs such as Th1/Th1 balance (5) and/or to explore the inter-relationship of these summary indices/joint distribution indicators in multiple regression analysis (6). See text for further details

Unfortunately, due to the inherent difficulty of applying these more sophisticated statistical approaches, researchers still use simple analytical approaches for multiple markers. Common strategies are to analyse each marker separately [16] or to include the battery of original markers simultaneously as predictors in multivariate regression models [5, 17, 18]. The flowchart shown in Fig. 2 illustrates the traditional multivariate regression approach for immune markers, neglecting any a priori knowledge about the existing underlying biological mechanisms or pathways. From a statistician’s perspective, this approach is vulnerable to serious methodological flaws that potentially can invalidate the results. The most frequent are: i) type I error inflation from multiple testings, ii) estimation problems due to multicollinearity or neglect of measurement error, and most importantly, iii) type II errors, i.e. missing true associations, because statistical analysis is based on immune markers that only reflect a small part of the variation of the underlying causal immunological mechanism.

In this paper, we present an extension of a previously described work [19], an analytical framework approach aimed at analysing multiple immune markers clustered at two functional levels by integrating existing knowledge about the underlying immunological mechanisms (Fig. 2). We illustrate the application of our approach to cytokine data from a large immuno-epidemiological study (SCAALA Salvador), which is aimed at investigating risk factors and immunological pathways for atopic diseases and asthma.

## Methods

This section consists of two parts. First, we describe the dataset used to illustrate the application of our analytical approach. Secondly, we present the theoretical framework for the analytical strategy.

### Study population

The SCAALA Salvador study enrolled 1445 children from Salvador in northeastern Brazil, a large urban center with a population of 2.8 million and a high prevalence of asthma symptoms such as wheezing (31 %) and atopy (38 %). The design of the study has been described in detail elsewhere [20]. Of the 1445 children originally included in the study, we obtained cytokine measurements for a subpopulation of 1006 children. Of those, we excluded 59 children with negligible mitogen response for all cytokines measured, likely due to laboratory errors. Of the remaining 957 children, we analysed data from 818 children with valid values for all 20 measurements (i.e. four cytokines and five immunological conditions).

### Immunological measurements

Venous blood was collected into heparinized tubes and cultured at a dilution of 1:4 in RPMI (Gibco, Auckland, New Zealand) supplemented with 10 mM glutamine (Sigma-Aldrich, St. Louis, MO, USA) and 100 μg/mL gentamicin (Sigma-Aldrich, St. Louis, MO, USA). We measured the production of four cytokines, IFN-γ, IL-5, IL-13, and IL-10, in supernatant fluids collected from whole blood cultures using commercially available antibody pairs and recombinant cytokine standards (BD Pharmingen, San Diego, CA, USA) by sandwich ELISA, according to the manufacturer’s instructions. Cytokine concentrations were determined by interpolation of standard curves. The detection limits (low/high) for each cytokine were 15.6/500 pg/mL, 62.5/4000 pg/mL, 18.5/300 pg/mL, and 31.3/500 pg/mL for IL-5, IL-13, IFN-γ, and IL-10, respectively. For each cytokine we obtained five different measurements for the different culture conditions. Whole blood cultures were started within 6 h of blood collection and were maintained in a humidified environment of 5 % CO2 at 37 °C for 24 h for detection of IL-10 and for 5 days for the detection of IL-13, IL-5, and IFN-γ in the presence of pokeweed mitogen (MITO, 2.5 μg/mL) (PWM; Sigma-Aldrich, St. Louis, MO, USA) media alone (spontaneous production/negative control (NC)), and the antigens *Ascaris lumbricoides* (ASC, 10 μg/mL), *Blomia tropicalis* (BLOM, 40 μg/mL), and *Dermaphagoides pteronyssinus* (DERM, 5 μg/mL), details of the cell culture stimulation experiments are described elsewhere (for ASC [21], for BLOM/DERM [20]). Cytokine measurements below detection limits were considered “non-responders” and assigned a cytokine concentration equal to the lower detection limit, while measurements above the detection limit were assigned a concentration equal to the upper detection limit. Children with detectable levels of cytokines (i.e. responders) were assigned to ordered categories with cut-offs based on median or tertiles for each cytokine and culture condition. IL-5 and IL-13 responses to mitogen, for which there was a higher proportion of responders, were classified into four categories (non-, low, intermediate, and high responders). All other measures were classified into three categories (non-, low, and high responders). We measured four different specific IgEs (sIGE) specific to *Dermaphagoides pteronyssinus*, *Blomia tropicalis*, *Periplaneta americana*, and *Blatella germanica*) in serum according to the manufacturer’s instructions (Phadia Diagnostics AB, Uppsala Sweden).

### Software

All statistical analyses were conducted using the statistical software package STATA (StataCorp. 2011. *Stata Statistical Software: Release 13*. College Station, TX: StataCorp LP).

## Analytical framework for statistical analysis of multiple immune biomarkers

Figure 2 contrasts the traditional regression approach and the suggested framework approach. Both analytical approaches start with an outcome variable of interest, e.g. a surrogate endpoint or intermediate biomarker such as specific IgE and potential immunological predictor variables, such as cytokines. In the Additional file 1 we contrast results of applying both the traditional approach and the framework approach to a typical immunological outcome (sIgE); these are further compared in the discussion section. In the following section we describe the suggested framework step by step.

### Step 1: Grouping immune markers by using pre-existing knowledge

**Fig. 3:**
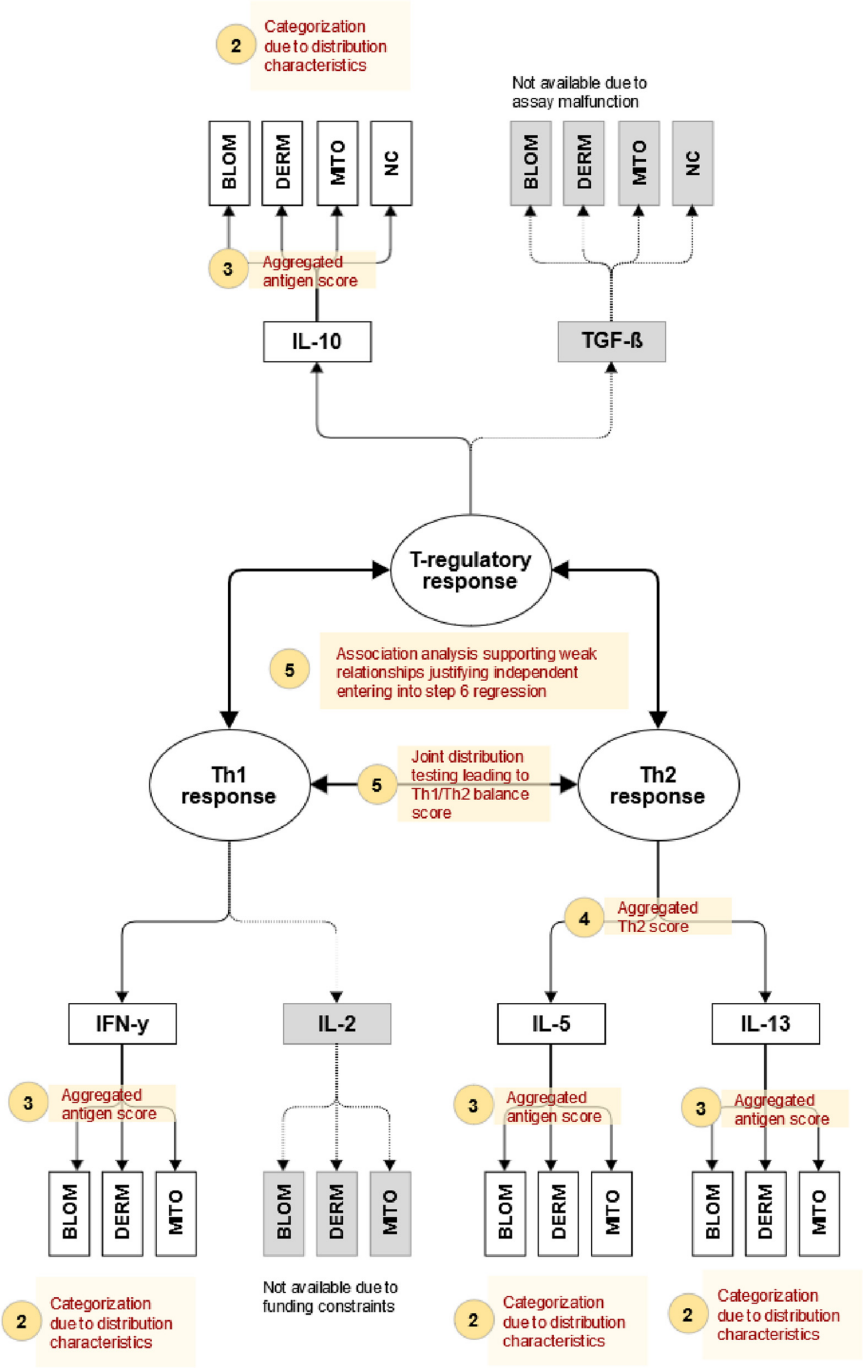
Conceptual model incorporating three inter-related immunological mechanisms (Th1-response, Th2-response, T-regulatory response) quantified by a battery of hierarchically clustered immune markers (multiple measurements of different cytokines). For each cytokine concentrations were determined from different cell cultures (ASC: *A. lumbricoides* specific response, BLOM: *B. tropicalis* specific response, DERM: *D. pteronyssinus* specific response*,* MITO: *pokeweed mitogen* response, NC: unstimulated *spontaneous response)*. The model incorporates a-priori knowledge about which cytokine relates to which specific mechanism, e.g. to use IFN-y as a marker for the Th1 response or to use IL-5 as a marker for the Th2 response. Further knowledge excludes the ASC measure assumed to reflect a more complex difficult to interpret immune response. The numbered bullets (2-5) correspond to the analytical steps illustrated in Fig. 2 (step 1 not shown) the legend to the bullet briefly summarizes the analytical step

The objective of the first step is to maximize the use of existing immunological knowledge (Figs. 1 and 2, step 1). To achieve this in immuno-epidemiological studies, researchers should group the immune markers according to a conceptual model that graphically represents the proposed relationships of each immune marker to an underlying immunological mechanism and the inter-relationship between these. Often a multilevel causal framework is necessary with multiple markers for each level. In allergy research, immunologists typically classify cytokines according to distinct types of immune responses (Th1 response, Th2 response, or regulatory (T-Reg) response). Further they obtain for each cytokine multiple measurements under different immunological conditions (e.g. from antigen-, mitogen- or non-stimulated cell culture experiments). In the multilevel context we consider level 1 as the multiple markers for a particular cytokine and level 2 as the different cytokine relating to a larger mechanism (e.g. IL-5 and IL-13 are both Th2 cytokines). As an example, the two-level conceptual framework for the cytokine data from the SCAALA study is shown in Fig. 3. We will discuss this framework in detail later in the results section.

### Step 2: Data exploration, data recoding

The objective of the second step is to examine the statistical properties of each measurement (Fig. 2, step 2a) and to employ both meaningful and statistically appropriate transformations and recoding (Fig. 2, step 2b). If this cannot be achieved, researchers may decide to omit the entire measurement; in SCAALA, for example, the TGF-ß measures could not be used due to assay malfunction. Empirically, the best approach is to assume that none of the data will follow a perfect normal distribution but will instead suffer from various violations of distributional assumptions, such as extreme skewness, non-detections, missing data, ceiling effects, or bimodal distributions. For specific distributional forms, data transformations can be used to achieve approximate normal distributions [6]. If data are truncated, for example by the “detection limits” of the assay, approaches such as the Kaplan-Meier method (for censored data) or the generalized Wilcoxon test may be applied to use this data for epidemiological association testing [10]. If theoretically justified, a simpler and more straightforward approach is to recode the data into distinct response categories (e.g. no, low, or high response). Cut points for defining response categories must be carefully chosen. However, unfortunately, in contrast to other areas of medical research such agreed cut-off values for immunological experiments often do not exist. In this case, cut points for categorisation should be based on characteristics of the distribution of the measurements, such as quantiles (e.g. median, tertiles, or quartiles). We strongly suggest refraining from bias that introduces oversimplification, e.g. categorising normally distributed continuous data into dichotomous categories such as low and high responders.

### Step 3: Intramarker (intragroup) analysis

Having arrived at statistically usable operationalisations of individual measurements in step 2, the objective of this step is to study the associative patterns within a level 1 immunological variable (Fig. 2, step 3a) and to search for further meaningful and statistically justified aggregation to summary scores (Fig. 2, step 3b). For example, association analysis of different stimulation methods for the same cytokine in culture experiments may suggest deriving one or more stimulation aggregate scores. Table 1 provides an overview of statistical methods for interdependence analysis and data aggregation of immunological markers, depending on the properties of the measurement identified in step 2. A good overview of the specific choice of methods is presented in our previously published guide for immunologists [19], while other authors provide details about factor analytic methods for continuous variables [22] or correspondence techniques for binomial, nominal, or ordinal variables [23]. As this step is crucially important, researchers should always first visually explore bivariate associations using contingency tables, correspondence bi-plots, or scatter plots (see examples from the present study in the Additional file 1).

**Table 1:** Statistical approaches for interdependence analysis of immunological markers dependent on the scale of measurement

Scale of measurement	Bivariate methods	Multivariate methods
Binomial (e.g. positive, negative)	Contingency table; tests: Chi-square or Fisher’s exact test; association measure: phi coefficient, Yule’s Q	Multilayer contingency table, classification trees
Nominal (e.g. Th1, Th2, or T-Reg)	Contingency table; tests: chi-square or Fisher’s exact test; association measure: contingency coefficient	Multilayer contingency tables, correspondence analysis, classification trees
Ordinal (e.g. low, medium, high)	Contingency table; tests: chi-square or Fisher’s exact test, tau test; association measure: Spearman-Rank correlation, Kendall’s Tau or Goodman and Kruskal’s γ	Multilayer contingency tables, correspondence analysis, classification trees
Continuous (non-normal distributed)	Scatter Plots; test: Spearman-Rank Correlation, criteria: Kendall’s Tau or Goodman and Kruskal’s γ	Factor analytic techniques: e.g. principal component analysis
Continuous (normal distributed)	Scatter Plots; test and association measure: Pearson correlation coefficient	Factor analytic techniques: e.g. principal component analysis

Rules of thumb for quantifying the strength of association based on the magnitude of association measures (e.g. Goodman and Kruskal’s γ): no association: 0 < |γ| < = 0.25, weak: 0.25 < |γ| < 0.50, moderate: 0.50 < |γ| < = 0.75, strong: |γ| > 0.75

The most important part of step 3 is deciding upon the potential aggregation of measurements to level 1 scores, e.g. whether measurements from various stimulation experiments with one cytokine read-out may be aggregated to an overall cytokine response score or not. We suggest the following criteria (see legend of Table 1 for decision cut-offs):

1. *Strong but simple positive association* patterns (i.e. high correlation or strong correspondence for categorical data) between a set of immune markers indicate the presence of a major underlying immunological mechanism. In this case, measurements should be combined using simple aggregation functions, such as *the average response* (for continuous data) or *the maximum response* (for categorical or ordinal data).
2. If multivariate correlation or correspondence analysis identifies *weak to moderate* associations or a *more complex association pattern* (e.g. markers with heterogeneous strengths of association to one or more underlying components), investigators should seek to reduce the multidimensional data to an immunologically meaningful factor solution. The resulting summary scores are weighted averages of the original markers, with factor loadings representing the weights.
3. If no *meaningful pattern* is observed, investigators should interpret the measures as

“immunologically independent”, with each measure likely to represent different underlying immunological phenomena. As a general rule, the aggregation of such independent markers to simple summary scores (e.g. the *sum of response)* should be avoided because information is lost; the interpretation of aggregated independent markers is more complex.

### Step 4: Intermarker analysis

If the data are conceptually structured in multiple levels, e.g. an immunological mechanism (level 2) being operationalised by responses of different cytokines, which in turn were obtained from measurements during multiple stimulation assays (level 1), the analytical strategy of step 3 should be repeated on the obtained aggregate scores from step 3. The objective again is to explore association patterns (Fig. 2, step 4a) allowing to further aggregate the data to immunological functional indices (or aggregated immunological scores) (Fig. 2, step 4b). For example, immunologists know that both IL-5 and IL-13 are produced by Th2 cells and thus show a strong association pattern due to this common mechanism (Th2-related immune response). In this case aggregating the antigen-specific summary scores for IL-5 and IL-13 (derived from step 3) to a Th2 summary score may be justified.

### Step 5: Interdependence or dependence analysis among the immunological summary indices

This step aims to explore the inter-relationship among the aggregated summary scores (Fig. 2, step 5a), either to elucidate possible interdependence or to perform dependence analysis. If there is no clear a priori hypothesis with respect to the direction of the relationship, the analytical approaches from steps 3 and 4 are repeated. In contrast, if investigators a priori assume an underlying mechanism, the assumptions about the direction of this mechanism should be incorporated in the model. For example, if researchers assume that T-Reg responses affect the Th2 response (and not vice versa), a regression model using the Th2 score as the dependent variable and T-Reg score as the independent (predictor) variable may be warranted (Fig. 2, step 5b). Further details describing the differences between dependence and interdependence analysis are described elsewhere [19].

### Step 6: Dependence analysis based on the immunological summary scores

The final step aims to relate the immunological summary scores derived in steps 1 to 5 to outcomes typically observed in immunological studies (e.g. clinical outcomes like skin prick test, asthma symptoms, or intermediate immune markers such as plasma sIgE concentrations) (Fig. 2, step 6).

## Results

To illustrate the application of the analytical framework we used data from 818 children with valid measurements of 20 immune markers, namely concentrations of four different cytokines (IL-10, IL-5, IL-13, and IFN-γ) obtained under five different stimulation experiments.

### Step 1: Grouping of variables using pre-existing knowledge

Figure 3 is a graphical representation of the pre-conceived relationships at the design of the study. The bullets refer to the steps of the analytical approach; step 1 is not shown. Conceptually, we consider the measurements to be hierarchically clustered in a two-level structure. At level 1 we consider for each cytokine the measurements obtained from the five stimulation experiments, and at level 2 we consider three inter-related latent immunological constructs: 1) an immune regulatory network, which is viewed as being interrelated with 2) Th1 responses and 3) Th2 immune responses. The regulatory response is quantified by concentrations of IL-10 and TGF-ß, the Th1 response by concentrations of IFN-γ and IL-2, and the Th2 response by concentrations of IL-5 and IL-13. In the present study, data from TGF-ß measurements were unusable due to assay malfunction and IL-2 measurements were not available due to funding constraints. We categorised the level 1 measurements into four groups. The first group includes the mite-specific responses *B. tropicalis (BLOM) and D. pteronyssinus (DERM),* which we assume a priori as highly immunologically related. In the second group, we place the responses to *A. lumbricoides* (ASC), which we expect to reflect a distinct and more complex helminth-induced immune response. In the third group, we consider the mitogen responses (MITO) presumed to reflect the maximum possible response, and in the fourth group the spontaneous non-stimulated cytokine responses. Further details on our a priori hypotheses about the presumed immunological mechanisms underlying the different cytokine measurements are presented in the legend to Fig. 3.

### Step 2: Data exploration, data recoding

Box plots visualising the distribution of the original data are shown in the (Additional file 1: Figure S1). We observed non-detects for all 20 cytokine measurements in considerable proportions of the participants (range 2.6 % to 97.9 %). Moreover, exploratory data analysis considering the cytokine concentration of responding individuals, including histograms and distributional plots (data not shown), suggested severe deviations from normality. Both observations indicated the necessity for a unifying ordinal categorisation of the data with the objective of retaining maximum possible information. We thus decided to pursue a three-category approach, except for mitogen responses of IL-5 and IL-13, where four categories were warranted. In the absence of an agreed immunological cut-off, we classified non-detects as non-responders, below-median measurements of detectable quantities as low responders, and above-median measurements as high responders. For mitogen responses of IL-5 and IL-13, detectable measurements were grouped using tertiles as cut-offs (low, intermediate, and high responders). Additional file 1: Table S1 provides the distributions of the recoded categorical data. As expected, cytokine response rates were higher in mitogen-stimulated cultures (78 % to 97 % responders) compared to antigen-specific cultures (3 % to 23 %) and unstimulated cultures (6 % to 35 % responders).

### Step 3: Intramarker (intragroup) analysis

This step aimed to detect new or confirm a priori hypothesized associations at level 1 (associations within the multiple measurements of the same cytokine). Table 2 shows a summary of this analytical step, displaying for each cytokine and all pairs of bivariate comparisons the association measure (Goodman and Kruskal’s-γ) and the *p*-value. To further illustrate the analyses conducted in this analytical step, we show detailed results for one cytokine (IL-13) in the (Additional file 1: Table S2 and Figure S2). The interdependence pattern identified among the five multiple measurements show that these measures quantify distinct immunological phenomena: the specific responses to different antigens, the non-specific response (MITO), and the unstimulated spontaneous immune response (NC). In more detail, we observed a very strong positive association between the mite-specific responses (BLOM, DERM) for IFN-γ, IL-5, and IL-13 (with γ ranging from 0.73 to 0.85); for IL-10 the correlation was substantially lower (γ = 0.35). In contrast, the mite-specific responses (BLOM, DERM) showed substantially weaker associations with helminth-specific responses (ASC), with the exception of IL-13, where all three antigen measures were highly related. This finding corroborates our a priori hypothesis to consider ASC as a separate immunological measure in any further analyses. However, we decided to aggregate the DERM and BLOM measures for each cytokine to a summary scale quantifying the overall mite-induced response (further called the ANTI scale) by considering the highest achieved response for either antigen. The distribution of this measure is shown in Table 3. Further, we found that for all cytokines the spontaneous (NC) and mitogen responses (MITO) showed absence of association (or inconsistent heterogeneous association patterns) with the antigen-specific measures and should thus be considered as separate scales in any further analyses.

**Table 2:** Summary of bivariate association analyses between each culture condition for each cytokine using data collected from 818 children in SCAALA Salvador

Cytokine	Measure	ASC	BLOM	DERM	MITO
IFN-γ	BLOM	**γ** = 0.21,	**-**	**-**	**-**
		P = 0.144			
	DERM	γ = 0.77***,	γ = 0.73**,	**-**	**-**
		**P = <0.001**	**P = <0.001**		
	MITO	γ = -0.24,	γ = 0.26*,	γ = 0.09,	**-**
		P = 0.667	**P = 0.016**	P = 0.653	
	NC	γ = 0.29*,	γ = 0.05,	γ = 0.11,	γ = -0.84***,
		P = 0.242	P = 0.593	P = 0.412	**P = <0.001**
IL-5	BLOM	γ = -0.05,	**-**	**-**	**-**
		P = 0.585			
	DERM	γ = 0.35*,	γ = 0.94***,	**-**	**-**
		P = 0.057	**P = <0.001**		
	MITO	γ = 0.26*,	γ = -0.21,	γ = 0.19,	**-**
		**P = 0.047**	P = 0.234	P = 0.568	
	NC	γ = 0.38*,	γ = 0.61**,	γ = 0.76***,	γ = 0.09,
		**P = <0.001**	**P = <0.001**	**P = <0.001**	P = 0.405
IL-13	BLOM	γ = 0.83***,	**-**	**-**	**-**
		**P = <0.001**			
	DERM	γ = 0.83***,	γ = 0.85***,	**-**	**-**
		**P = <0.001**	**P = <0.001**		
	MITO	γ = 0.47*,	γ = 0.28*,	γ = 0.52**,	**-**
		**P = <0.001**	P = 0.016	**P = <0.001**	
	NC	γ = 0.43*,	γ = 0.70**,	γ = 0.45*,	**γ = 0.09,**
		**P = <0.001**	**P = <0.001**	**P = <0.001**	**P = 0.005**
IL-10	BLOM	γ = 0.28*,	**-**	**-**	**-**
		P = 0.510			
	DERM	γ = 0.69**,	γ = 0.35*,	**-**	**-**
		**P = <0.001**	**P = <0.001**		
	MITO	γ = 0.26*,	γ = 0.93***,	γ = 0.43*,	**-**
		P = 0.326	**P = <0.001**	**P = <0.001**	
	NC	γ = -0.03,	γ = -0.95***,	γ = -0.35*,	γ = -0.96***,
		P = 0.468	**P = <0.001**	**P = 0.008**	**P = <0.001**

ASC: response in cell culture stimulated by *A. lumbricoides,* BLOM: response in cell culture stimulated by *B. tropicalis,* DERM: response in cell culture stimulated by *D. pteronyssinus,* MITO: response in cell culture stimulated by *pokeweed mitogen*, NC: response in nonstimulated cell culture; γ: Goodman and Kruskal’s γ, P: *P*-value of chi-square test of independence; Strength of association: * … weak (0.25 < |γ| < =0.5)

** … moderate (0.50 < |γ| < =0.75); *** … strong (0.75 < |γ| < =1); Significant estimates (*P*<0.05) are shown in bold

**Table 3:** Distribution of aggregated summary scales for the overall dust-mite antigen-specific response using data collected from 818 children in SCAALA Salvador

Cytokine	IFN-γ		IL-5		IL-13		IL-10	
	n	%	n	%	n	%	n	%
no response	604	73.8	796	97.3	652	79.7	22	2.7
low response	94	11.5	11	1.3	77	9.4	152	18.6
high response	120	14.7	11	1.3	89	10.9	644	78.7
Total	818	100.0	818	99.9	818	100.0	818	100.0

Maximum immune responses observed in cell cultures stimulated by *B. tropicalis or D. pteronyssinus*

### Step 4: Intermarker analysis

In this step, we conducted an intercytokine analysis examining the association patterns between any pair of cytokines on three different scales (ANTI, MITO, and NC) (Table 4). As expected, we observed for the two Th2-related cytokines IL-5 and IL-13 positive associations on both antigen and mitogen scales (γ = 0.58, *P* = 0.001 for ANTI, and γ = 0.62, *P* < 0.001 for MITO), but no association on the spontaneous scale (γ = -0.22, *p* = 0.451 for NC). These results were corroborated by correspondence analysis (Additional file 1: Figure S3). Given this moderate positive association, we calculated a Th2 summary score by considering the highest observed response category of either Th2-related cytokine (maximum response of IL-5 and IL-13). We calculated the Th2 score for both the antigen and mitogen scale.

**Table 4:** Summary of result of intercytokine analysis using data collected from 818 children in SCAALA Salvador

Measure	Cytokine	IL-5	IL-13	IL-10
ANTI	IFN-γ	γ = -0.36*, P = 0.669	γ = 0.12, P = 0.324	γ = 0.33*, P = 0.011
	IL-5		γ = 0.58**, P = 0.001	γ = 0.06, P = 0.242
	IL-13			γ = -0.04, P = 0.648
MITO	IFN-γ	γ = 0.53**, P < 0.001	γ = 0.65**, P < 0.001	γ = 0.46*, P < 0.001
	IL-5		γ = 0.62**, P < 0.001	γ = 0.27*, P < 0.001
	IL-13			**γ = 0.19, P = 0.008**
NC	IFN-γ	γ = 0.18, P = 445	γ = 0.09, P = 0.095	γ = 0.04, P = 0.500
	IL-5		γ = -0.22, P = 0.451	γ = -0.39*, P = 0.620
	IL-13			γ = 0.07, P = 0.484

ANTI: Maximum immune response observed in cell cultures stimulated by *B. tropicalis* or *D. pteronyssinus,* MITO: response in cell cultures stimulated by *pokeweed mitogen*, NC: response in non-stimulated cell cultures; γ: Goodman and Kruskal’s γ, P: *P*-value of chi-square test of independence, significant associations are shown in bold; Strength of association: * … weak (0.25 < |γ| < =0.5), ** … moderate (0.50 < |γ| < =0.75)

*** … strong (0.75 < |γ| < =1); Significant estimates (*P*<0.05) are shown in bold

Considering the antigen scale, the Th1 response (IFN-γ) showed independence of the Th2 response (IL-5, IL-13) but positive association with the regulatory response (IL-10). Further, we found that all four cytokines showed positive association patterns on the mitogen scale but no association on the spontaneous response. As explained above, the present study yielded only one cytokine each for the regulatory response and the Th1 response, allowing the intermarker aggregation step for Th1 and T-Reg constructs to be skipped.

For any further analysis steps, we considered two scales (antigen and mitogen) for Th1 and Th2 responses and three scales (antigen, mitogen, and spontaneous) for T-Reg responses. In addition, since one of the main hypotheses about the immunological mechanisms underlying atopic diseases and asthma relates to the balance of Th1 and Th2 cytokines, we cross-tabulated the Th1 and Th2 aggregated scores considering the joint Th1-Th2 response. We grouped the data into a Th1/Th2 balance score with higher values referring to Th2 skewness: 0:Th1 negative and Th2 negative, 1:Th1 positive and Th2 negative, 2: Th1 positive and Th2 positive, and 3: Th1 negative and Th2 positive. The balance indicator was calculated for both antigen and mitogen scale. Table 5 shows the distributions of the final immunological summary scores that will be considered in the next analysis steps.

**Table 5:** Distributions of final immunological summary scales derived from cytokine data collected from 818 children in SCAALA Salvador

T-Reg response	ANTI		MITO		NC	
	n	%	n	%	n	%
no response	22	2.7	21	2.6	749	91.6
low response	152	18.6	121	14.8	36	4.4
high response	644	78.7	676	82.6	33	4.0
Th1 response	ANTI		MITO			
	n	%	n	%		
no response	604	73.8	71	8.7		
low response	94	11.5	137	16.7		
high response	120	14.7	610	74.6		
Th2 response	ANTI		MITO			
	n	%	n	%		
no response	642	78.5	99	12.1		
low response	79	9.7	162	19.8		
intermediate response	-	-	218	26.7		
high response	97	11.8	339	41.4		
TH1/TH2 balance	ANTI		MITO			
	n	%	n	%		
no TH1 resp./no TH2 resp.	481	58.8	36	4.4		
TH1 resp./no. TH2 resp.	161	19.7	63	7.7		
TH1 resp./TH2 resp.	53	6.5	684	83.6		
no TH1 resp./TH2 resp.	123	15.0	35	4.3		

ANTI: Maximum immune response observed in cell cultures stimulated by *B. tropicalis* or *D. pteronyssinus*, MITO: response in cell cultures stimulated by *pokeweed mitogen*, NC: response in non-stimulated cell cultures

### Step 5: Interdependence or dependence analysis among the immunological summary scores

In this step, we conducted an exploratory interdependence analysis among the summary scores Th1, Th2, Th1/Th2 balance, and T-Reg. A summary of the results is presented in Table 6; plots of correspondence analysis are displayed in the (Additional file 1: Figure S5). Three important findings have emerged: i) Th1/Th2 balance on the antigen scale presented as independent of T-Reg, both for spontaneous and mitogen scales (NC:γ = 0.20, *P* = 0.387, MITO : γ = 0.22, P = 0.313), ii) a weak association between Th2 response and T-Reg (NC, γ = 0.30, *P* = 0.008); iii) for antigen responses no association between Th1 and Th2 (ANTI, γ = 0.06, P = 0.248), no association between Th2 and T-Reg (ANTI, γ = -0.02), and small association between Th1-Th2 balance and T-reg (ANTI, γ = 0.14, *P* = 0.030); iv) considering the mitogen responses all four summary scores showed positive associations. The important consequence of these results for step 6 dependency analysis is to consider T-Reg and Th1/Th2 balance as independent immunological variables. We consider the antigen scale as the primary measure to quantify Th1/Th2 balance and the spontaneous scale of T-Reg to quantify the immune regulatory mechanism.

**Table 6:** Results of interdependence analysis among the immunological summary scores

Immune response		Th2 response		T-Reg response		
	Measure	ANTI	MITO	ANTI	MITO	NC
Th1 response	ANTI	γ = 0.06, P = 0.248	γ = 0.25*, P < 0.001	γ = 0.33*, P = 0.011	γ = 0.14, P = 0.504	γ = 0.11, P = 0.302
	MITO	γ = -0.01, P = 0.723	γ = 0.59**, P < 0.001	γ = 0.42*, P < 0.001	γ = 0.46*, P < 0.001	γ = 0.26*, P = 0.021
Th2 response	ANTI	-	**-**	γ = -0.02, P = 0.509	γ = -0.13, P = 0.458	γ = 0.30*, P = 0.008
	MITO	-	**-**	γ = 0.36*, P < 0.001	γ = 0.26*, P < 0.001	γ = 0.10, P = 0.280
Th1/Th2 balance	ANTI	-	-	**γ = 0.14, P = 0.030**	γ = -0.01, P = 0.437	γ = 0.20, P = 0.387
	MITO	-	**-**	γ = 0.39*, P < 0.001	**γ = 0.17, P < 0.001**	γ = 0.22, P = 0.313

ANTI: Maximum immune response observed in cell cultures stimulated by *B. tropicalis* or *D. pteronyssinus*, MITO: response in cell cultures stimulated by *pokeweed mitogen*, γ: Goodman and Kruskal’s γ, P: *P*-value of chi-square test of independence, significant associations shown in bold; strength of association: * … weak (0.25 < |γ| < =0.5)

** … moderate (0.50 < |γ| < =0.75), *** … strong (0.75 < |γ| < =1); Significant estimates (*P*<0.05) are shown in bold

### Step 6: Dependence analysis based on the immunological summary scores

Finally, we illustrate how the immunological summary scores derived in steps 1 to 5 can be used to describe the association with an important immunological outcome (sIgE). We compare the results of our framework approach with a classical stepwise regression approach using the log-transformed raw cytokine (Fig. 2). Detailed results of both approaches are shown in the (Additional file 1: Table S3). In summary, the traditional stepwise regression approach revealed five significant parameters. We found a significant strong positive effect of IL-5 (*B. tropicalis*), a 10-fold smaller positive effect for IL-5 (*D. pteronyssinus),* a 60-fold smaller positive effect of IL-5 (MITO) and IL-10 concentrations (*D. pteronyssinus),* and a 20-fold smaller negative association with IFN-γ (*B. tropicalis*). All other parameters failed to retain significance.

By contrast, the framework approach results in the aggregated immunological summary scores that were included in a multivariate regression model as predictors for sIgE, namely the dummy-coded Th1/Th2 balance score (antigen scale) and the dummy-coded T-Reg score (spontaneous scale). Since we do not assume any effect of Th1 on sIgE, we recoded the Th1/Th2 balance score by pooling the first two categories (Th1-/Th2- and Th1+/Th2-). Moreover, since we expect a downregulating effect of strong IL-10 responses we dichotomize the T-Reg score (high vs. low or no response). The detailed results are shown in the (Additional file 1: Table S2, B). Briefly, compared to the reference category (absence of Th2 response) the model showed a strong significant increase of sIgE in the case of a skewed Th2+ response (Th1-/Th2+: ß = 0.53, *P* = 0.009) and a non-significant, substantially smaller increase induced by a balanced Th2 response (Th1+/Th2+: ß = 0.30, P = 0.304). Additionally, the model reveals a negative but non-significant effect of a high spontaneous IL-10 response (ß = -0.38, *P* = 0.298). We also investigated the inclusion of interaction terms between Th1/Th2 balance and T-Reg that did not show any significant effects. We will not discuss this dependence analysis any further here; the detailed effects of cytokine pattern on sIgE and other endpoints observed in SCAALA, such as the skin prick test or clinical outcomes like allergy or asthma symptoms, will be examined in future work.

### Traditional regression approach

In contrast, the application of the traditional stepwise regression to the original cytokine measures as described in Fig. 2 (left side) yielded results that were not straightforward to interpret. While finding a strong positive effect of IL-5 and a negative effect of IFN-γ (both specific to *B. tropicalis)* was consistent with the hygiene hypothesis, the interpretation of other parameters in the final model was difficult. For example, the much lower effect of IL-5 (*D. pteronyssinus*) compared to IL-5 (*B. tropicalis*) or the statistically significant association with high IL-10 (DERM) might have arisen from multicollinearity or other peculiarities, such as deviation from the multivariate normal distribution of this data (more details, Additional file 1).

## Discussion

In the present work, we present an approach for the statistical analysis of multiple, often related immunological markers that capitalizes on a priori existing knowledge and insight into immunological mechanisms. We illustrated the proposed approach using a dataset from a large immuno-epidemiological study (SCAALA Salvador) conducted to investigate the potential immunological mechanisms underlying atopic diseases and asthma. The immune markers collected in this study included multiple measurements of different cytokines obtained under different whole blood culture conditions.

The application of our approach to the cytokine data collected in SCAALA Salvador suggested Th1/Th2 skewness as the most relevant predictor for elevated sIgE levels, rather than the Th2 response itself. Further, the approach suggested a potential independent down-regulating effect of strong T-regulatory spontaneous responses. In contrast, the traditional stepwise regression approach using the full dataset and all original cytokine measures yielded results that were difficult to interpret (e.g. a statistically significant association of sIgE and the IL-10 release in response to the DERM mite antigen).

What is the research implication of this approach and why it might prove superior to the traditional statistical approach? The suggested approach is still based on common statistical concepts such as the idea of latent variables. However, the stepwise implementation is less data driven because each analytical step is guided by a conceptual model that specifies the presumed underlying immunological mechanism. Conceptually, the approach resembles the work employed by empirical scientists from other areas, e.g. the social sciences, aimed to investigate complex phenomena involving concepts that are not directly observable (e.g. intelligence or socioeconomic status). Approaches like structural equation modelling are frequently used to implement the underlying concepts by using measurement models based on multi-layered indicator variables.

In the proposed approach, we invite immuno-epidemiologists to follow this well-developed approach of investigation including the idea of latent variables. Our step 1 postulates to order or cluster measurements according to a preconceived conceptual model. This should reflect the best available knowledge at the time of conducting the analysis. For example, in the present study, researchers sought to characterize the T-regulatory response by obtaining concentrations of IL-10 and TGF-ß in stimulated and non-stimulated cell cultures. Our step 2 deals with the problem that rarely immunological data follow a normal distribution. Researchers need to make decisions how to control the statistical peculiarities and to reduce the data, if necessary, e.g. to binary or ordered variables that are amenable to downstream statistical procedures. In the present example, the non-normal distribution of the data (see Additional file 1) required to employ different categorization levels. Step 3 is an analogy to forming scales from individual questionnaire items. In theory, such aggregated scores may yield less measurement error and provide information more related to the underlying construct. In the present study, the IL-5, IL-13, IFN- γ and IL-10 aggregated antigen scores reflect this step. Step 4 allows further aggregation, just like different scales may aggregate to a well-being index in psychosocial research. In the present example, the IL-5 and IL-13 scores are further aggregated to an overall aggregated Th2 score. Like in the mentioned psychosocial example both step 3 and step 4 require active and thoughtful participation of the researcher to consider the possible content validity of score or index formation in the context of concurrent immunological knowledge. Once the researcher has arrived at aggregated non redundant measures that reflect the underlying construct simple statistical procedures can be employed to quantify the relationship between the constructs (e.g ratios or cross tables examining the joint distribution). In the present example this step 5 involved constructing a Th1/Th2 balance score just by considering the joint distribution of Th1 and Th2 responses. In the final step, we proposed simple regression models aimed to relate the immunological summary constructs to outcomes of interest are the objective of the final step 6 (e.g. regressing sIgE levels onto Th1/Th2 balance and T-regulation). Results from these models are easier to interpret than models using the original markers because they better quantify the impact of the potentially causing mechanisms on the outcomes of interest.

The strengths of this approach simultaneously bears the possible limitations. The strength at each step is to include the best available immunological knowledge in decision making about statistical procedures. For example, in step 2 decisions must be made to deal with missing data, with non-normally distributed data, whether the aggregated score has binominal (response yes-no) or ordinal characteristics (no-low-medium-high response). The limitation of the approach is that each step may introduce potential bias (e. g. from arbitrary choices of cut-offs). However, if in future reports using this approach every step is carefully documented, together with providing the raw data in appendices or journal repositories, other researchers may choose to repeat the analysis using different aggregation modalities or to introduce new conceptual models as scientific insight progresses. Such informed, transparent and conscious analytical process may contribute to a better understanding of complex immunological mechanisms.

## Conclusions

We suggest a systematic analytical approach for complex immuno-epidemiological data that capitalizes on a framework incorporating pre-existing knowledge about the underlying immunological phenomena. Our approach mimics the method of latent variable approaches such as principal components analysis, latent class analysis or structural equation modelling. However, with less harsh data assumption our analytical strategy circumvents peculiarities often encountered in real data such as violation of distributional assumptions or multicollinearity. The proposed approach may assist applied researchers in the area of immuno-epidemiology to quantify the immunological mechanisms underlying immunologically mediated diseases, such as allergic disease or asthma. Using immunological summary scores that reflect distinct immunological concepts instead of the correlated original markers should substantially simplify data analysis in studies on relationships among non-immunological factors, immune responses, and disease.

## Ethics approval and consent to participate

The study was approved by the Brazilian National Ethical Committee in 2004, and written informed consent was obtained from the legal guardian of each study participant.

## Availability of data and material

The dataset supporting the conclusions of this article is available on request. Please contact the corresponding author per email.

## Additional file

Additional file 1:
**Table S1.** Distributions of recoded ordinal cytokine data (818 children from SCAALA Salvador). **Table S2.** Bivariate association analysis for IL-13 responsiveness produced under different culture conditions in vitro using data collected from 818 children in SCAALA Salvador. **Table S3.** Comparison of statistical approaches used to model the effect of cytokine concentrations on the outcome sIgE (maximum concentration of five antigens). **Figure S1.** Boxplots of cytokine concentrations (in pg/ml) obtained from different cell cultures (N=818 children from SCAALA Salvador). **Figure S2.** Intra-cytokine analysis: Correspondence pattern among IL-13 measures. **Figure S3.** Th2 inter-cytokine analysis: correspondence analysis biplots IL-5 vs. IL-13. **Figure S4.** Correspondence analysis biplots Th2 response and Th1/Th2 balance vs. T-Reg response. (DOCX 332 kb)

## Abbreviations

SCAALA: Research Programme “Social Changes Allergy and Asthma in Latin America”
IL: interleukin
IFN: interferon
PWM: pokeweed mitogen
MITO: mitogen induced response
ASC: *Ascaris lumbricoides*
BLOM: *Blomia tropicalis*
DERM: *Dermaphagoides pteronyssinus*
NC: spontaneous production (negative control)
sIgE: specific immunoglobulin E
T-Reg: T-regulatory cells
TGF-ß: transforming growth factor-ß
ANTI: overall mite-induced response
