# Supplementary material for: Applied immuno-epidemiological research: an approach for integrating existing knowledge into the statistical analysis of multiple immune markers
Source: BMC Immunol. 2016 May 20;17:11. doi: 10.1186/s12865-016-0149-9 (PMC4875650; doi:10.1186/s12865-016-0149-9)
Supplement: Additional file 1: — Table S1. Distributions of recoded ordinal cytokine data (818 children from SCAALA Salvador). Table S2. Bivariate association analysis for IL-13 responsiveness produced under different culture conditions in vitro using data collected from 818 children in SCAALA Salvador. Table S3. Comparison of statistical approaches used to model the effect of cytokine concentrations on the outcome sIgE (maximum concentration of five antigens). Figure S1. Boxplots of cytokine concentrations (in pg/ml) obtained from different cell cultures (N=818 children from SCAALA Salvador). Figure S2. Intra-cytokine analysis: Correspondence pattern among IL-13 measures. Figure S3. Th2 inter-cytokine analysis: correspondence analysis biplots IL-5 vs. IL-13. Figure S4. Correspondence analysis biplots Th2 response and Th1/Th2 balance vs. T-Reg response. (DOCX 332 kb) [file 12865_2016_149_MOESM1_ESM.docx]

**Additional file**

Table S1: Distributions of recoded ordinal cytokine data (818 children from SCAALA Salvador)

| Cytokine | ASC | | BLOM | | DERM | | MITO | | NC | |
| --- | --- | --- | --- | --- | --- | --- | --- | --- | --- | --- |
| IFN-γ | n | % | n | % | n | % | n | % | n | % |
| no response | 796 | 97.3 | 620 | 75.8 | 757 | 92.5 | 71 | 8.7 | 728 | 89.0 |
| low response | 12 | 1.5 | 95 | 11.6 | 31 | 3.8 | 137 | 16.7 | 45 | 5.5 |
| high response | 10 | 1.2 | 103 | 12.6 | 30 | 3.7 | 610 | 74.6 | 45 | 5.5 |
| IL-5 | n | % | n | % | n | % | n | % | n | % |
| no response | 712 | 87.0 | 810 | 99.0 | 801 | 97.9 | 183 | 22.4 | 765 | 93.5 |
| low response | 56 | 6.8 | 4 | 0.5 | 8 | 1.0 | 206 | 25.2 | 27 | 3.3 |
| intermediate response | - | - | - | - | - | - | 217 | 26.5 | - | - |
| high response | 50 | 6.1 | 4 | 0.5 | 9 | 1.1 | 212 | 25.9 | 26 | 3.2 |
| IL-13 | n | % | n | % | n | % | n | % | n | % |
| no response | 658 | 80.4 | 767 | 93.8 | 666 | 81.4 | 140 | 17.1 | 532 | 65.0 |
| low response | 78 | 9.5 | 25 | 3.1 | 71 | 8.7 | 211 | 25.8 | 141 | 17.2 |
| intermediate response | - | - | - | - | - | - | 231 | 28.2 | - | - |
| high response | 82 | 10.0 | 26 | 3.2 | 81 | 9.9 | 236 | 28.9 | 145 | 17.7 |
| IL-10 | n | % | n | % | n | % | n | % | n | % |
| no response | 780 | 95.4 | 24 | 2.9 | 628 | 76.8 | 21 | 2.6 | 749 | 91.6 |
| low response | 19 | 2.3 | 153 | 18.7 | 85 | 10.4 | 121 | 14.8 | 36 | 4.4 |
| high response | 19 | 2.3 | 641 | 78.4 | 105 | 12.8 | 676 | 82.6 | 33 | 4.0 |

Legend Table 1:

BLOM: response in cell culture stimulated by *B. tropicalis,* DERM: response in cell culture stimulated by D. pteronyssinus, ASC: response in cell culture stimulated by A. lumbricoides, MITO: response in cell culture stimulated by *pokeweed* *mitogen,* NC: response in non-stimulated cell culture

Table S2: Bivariate association analysis for IL-13 responsiveness produced under different culture conditions *in vitro* using data collected from 818 children in SCAALA Salvador.

1. BLOM vs. DERM (γ = 0.83, P = <0.001)

|  | DERM | | | | | | | |
| --- | --- | --- | --- | --- | --- | --- | --- | --- |
|  | no response | | low response | | high response | | total | |
| BLOM | n | % | n | % | n | % | n | % |
| no response | 652 | 85.0 | 64 | 8.3 | 51 | 6.7 | 767 | 100.0 |
| low response | 7 | 28.0 | 6 | 24.0 | 12 | 48.0 | 25 | 100.0 |
| high response | 7 | 26.9 | 1 | 3.9 | 18 | 69.2 | 26 | 100.0 |
| total | 666 | 81.4 | 71 | 8.7 | 81 | 9.9 | 818 | 100.0 |

1. BLOM vs. MITO (γ = 0.28, P = 0.016)

|  | MITO | | | | | | | | | |
| --- | --- | --- | --- | --- | --- | --- | --- | --- | --- | --- |
|  | no response | | low response | | intermediate response | | high  response | | total | |
| BLOM | n | % | n | % | n | % | n | % | n | % |
| no response | 134 | 17.5 | 201 | 26.2 | 221 | 28.8 | 211 | 27.5 | 767 | 100.0 |
| low response | 1 | 4.0 | 5 | 20.0 | 8 | 32.0 | 11 | 44.0 | 25 | 100.0 |
| high response | 5 | 19.2 | 5 | 19.2 | 2 | 7.7 | 14 | 53.9 | 26 | 100.0 |
| total | 140 | 17.1 | 211 | 25.8 | 231 | 28.2 | 236 | 28.9 | 818 | 100.0 |

1. DERM vs. MITO (γ = 0.52, P = <0.001)

|  | MITO | | | | | | | | | |  |
| --- | --- | --- | --- | --- | --- | --- | --- | --- | --- | --- | --- |
|  | no response | | low response | | intermediate response | | high response | | total | |  |
| DERM | n | % | n | % | n | % | n | % | n | % | |
| no response | 137 | 20.6 | 184 | 27.6 | 187.0 | 28.1 | 158 | 23.7 | 666 | 100.0 | |
| low response | 0 | 0.0 | 22 | 31.0 | 24.0 | 33.8 | 25 | 35.2 | 71.0 | 100.0 | |
| high response | 3 | 3.7 | 5 | 6.2 | 20.0 | 24.7 | 53 | 65.4 | 81.0 | 100.0 | |
| total | 140 | 24.3 | 211 | 64.8 | 231.0 | 86.6 | 236 | 124.3 | 818 | 100.0 | |

1. MITO vs. NC (γ = 0.09, P < 0.001)

|  | NC | | | | | | | |
| --- | --- | --- | --- | --- | --- | --- | --- | --- |
|  | no  response | | low response | | high response | | total | |
| MITO | n | % | n | % | n | % | n | % |
| no response | 83 | 59.3 | 31 | 22.1 | 26.0 | 18.6 | 140 | 100.0 |
| low response | 157 | 74.4 | 31 | 14.7 | 23.0 | 10.9 | 211 | 100.0 |
| high response | 140 | 59.3 | 39 | 16.5 | 57.0 | 24.2 | 236 | 100.0 |
| total | 380 | 193.0 | 101 | 53.4 | 106 | 53.6 | 587 | 100.0 |

Legend Table S2:

BLOM: response in cell culture stimulated by *B. tropicalis,* DERM: response in cell culture stimulated by D. pteronyssinus, MITO: response in cell culture stimulated by *pokeweed* *mitogen*, NC: response in non-stimulated cell culture; γ = *Goodman and Kruskal’s* γ

Table S3) Comparison of statistical approaches used to model the effect of cytokine concentrations on the outcome sIgE (maximum concentration of five antigens):

1. Stepwise regression approach (P(include)=0.05, P(exclude)=0.1) starting with full model including all cytokine measurements as predictors:

Full model (N=818, R²=0.068):

Parameter Coef. Std. Error t P>t [95% Conf. Interval]

IFN-γ (MITO) .0011610 .0009297 1.25 0.212 -.0006639 .0029859

IFN-γ (NC) .0022528 .0026494 0.85 0.395 -.0029478 .0074534

IFN-γ (DERM) .0011905 .0024662 0.48 0.629 -.0036506 .0060316

IFN-γ (BLOM) -.0036756 .0010435 -3.52 **0.000** -.0057238 -.0016273

IFN-γ (ASC) -.0042723 .0070158 -0.61 0.543 -.0180439 .0094992

IL-5 (MITO) .0008831 .0006096 1.45 0.148 -.0003135 .0020797

IL-5 (NC) .0316113 .0246051 1.28 0.199 -.0166872 .0799098

IL-5 (DERM) .0059320 .0031839 1.86 0.063 -.0003178 .0121817

IL-5 (BLOM) .0643179 .0212742 3.02 **0.003** .0225578 .1060779

IL-5 (ASC) .0002972 .0012151 0.24 0.807 -.002088 .0026823

IL-13 (MITO) .0000502 .0001056 0.48 0.635 -.000157 .0002574

IL-13 (NC) -.0002557 .0003055 -0.84 0.403 -.0008553 .000344

IL-13 (DERM) -.0001454 .0003738 -0.39 0.697 -.000879 .0005883

IL-13 (BLOM) -.0015155 .0007873 -1.92 0.055 -.003061 .0000299

IL-13 (ASC) .0006570 .0004929 1.33 0.183 -.0003105 .0016245

IL-10 (MITO) -.0016308 .0010186 -1.60 0.110 -.0036301 .0003686

IL-10 (NC) -.0021785 .0015819 -1.38 0.169 -.0052836 .0009266

IL-10 (DERM) .0012293 .0004651 2.64 **0.008** .0003163 .0021423

IL-10 (BLOM) .0001743 .0008350 0.21 0.835 -.0014648 .0018133

IL-10 (ASC) -.0013787 .0014063 -0.98 0.327 -.0041393 .0013819

Constant -.7626942 .5885359 -1.30 0.195 -1.917958 .3925693

Final model after stepwise algorithm (N=818, R²=0.054):

Parameter Coef. Std. Error t P>t [95% Conf. Interval]

IL-10 (DERM) .0012180 .0004300 2.83 **0.005** .0003739 .0020620

IL-5 (MITO) .0012063 .0004971 2.43 **0.015** .0002307 .0021820

IL-5 (BLOM) .0674111 .0208156 3.24 **0.001** .0265524 .1082699

IFN-γ (BLOM) -.0037125 .0009908 -3.75 **0.000** -.0056573 -.0017677

IL-5 (DERM) .0067589 .0029443 2.30 **0.022** .0009796 .0125382

Constant -.6892018 .1016693 -6.78 **0.000** -.8887674 -.4896361

Legend table S3: Regression coefficients reflect the change in log-transformed sIgE (unit: KU/l) per change in cytokine concentration (unit: pg/mL).

1. Framework approach including the immunological summary scores Th1/Th2 balance and T-Reg as predictors in the regression models (N=818, R²=0.009):

Parameter Coef. Std. Error t P>t [95% Conf. Interval]

Th1/Th2 balance (ANTI)

Th1+/Th2+ .3032749 .2946546 1.03 0.304 -.2750976 .8816473

**Th1-/Th2+ .5334773 .2029847 2.63 0.009 .1350422 .9319124**

T-Reg (NC) -.3817676 .3668745 -1.04 0.298 -1.1018990 .3383640

Constant -.5750811 .082135 -7.00 **0.000** -.7363024 -.4138598

Legend Table S3:

BLOM: response in cell culture stimulated by *B tropicalis,* DERM: response in cell culture stimulated by D pteronyssinus, MITO: response in cell culture stimulated by *pokeweed* *mitogen*, NC: response in non-stimulated cell culture; Parameter Th1+/Th2+: Effect of a balanced antigen specific Th2 response (Th1 is positive) vs. the reference category (no Th2 response); Parameter Th1+/Th2+: Effect of a skewed antigen specific Th2 response (Th1 is negative) vs. reference category (no Th2 response); Parameter T-Reg: Effect of a high T-Reg response (IL10 spontaneous) vs. reference category (no or low T-Reg response)

Figure S1: Boxplots of cytokine concentrations (in pg/ml) obtained from different cell cultures (N=818 children from SCAALA Salvador)

1. IFN-γ: B) IL-5:


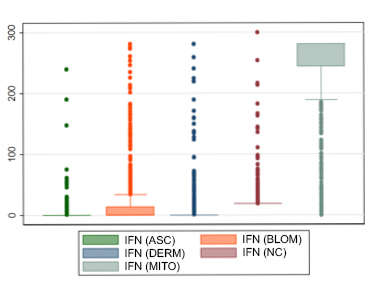

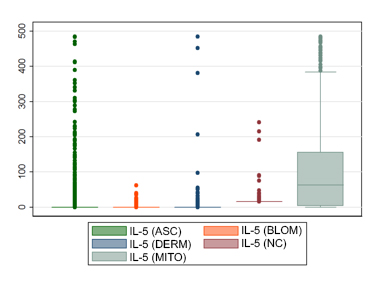


1. IL-13: D) IL-10:

**
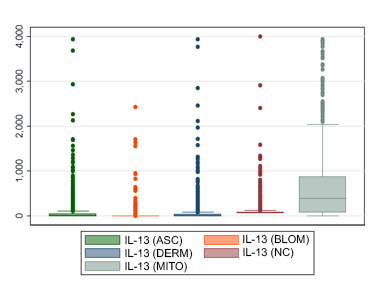

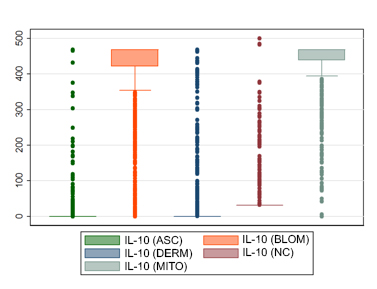
**

Legend Figure S1:

BLOM: response in cell culture stimulated by *B tropicalis,* DERM: response in cell culture stimulated by D pteronyssinus, MITO: response in cell culture stimulated by *pokeweed* *mitogen*, NC: response in non-stimulated cell culture

Figure S2: Intra-cytokine analysis: Correspondence pattern among IL-13 measures:

IL-13 (MITO)

IL-13 (DERM)

IL-13 (BLOM)

IL-13 (ASC)

Figure S3: Th2 inter-cytokine analysis: correspondence analysis biplots IL-5 vs. IL-13.

1. Antigen-specific response:

IL-13 (ANTI)

IL-5 (ANTI)

1. Mitogen response:

IL-13 (MITO)

IL-5 (MITO)

Figure S4: Correspondence analysis biplots Th2 response and Th1/Th2 balance vs. T-Reg response

1. Th2 (ANTI) vs. T-Reg (NC): B) Th2 (ANTI) vs. T-Reg (MITO):

T-Reg2

T-Reg1

Th2 (ANTI)

Th2 (ANTI)

C) Th2 (ANTI) vs. T-Reg (ANTI): D) Th1/Th2 balance (ANTI) vs. T-Reg (NC):

T-Reg4

Th2 (ANTI)

T-Reg1

Th1/Th2 balance

E) Th1/Th2 balance (ANTI) vs. T-Reg (MITO): F) Th1/Th2 balance (ANTI) vs. T-Reg (ANTI):

T-Reg4

T-Reg2

Th1/Th2 balance

Th1/Th2 balance
